# COPD patients prescribed inhaled corticosteroid in primary care: time for re-assessment based on exacerbation rate and blood eosinophils?

**DOI:** 10.1186/s12931-021-01651-w

**Published:** 2021-02-12

**Authors:** Osman Savran, Nina Godtfredsen, Torben Sørensen, Christian Jensen, Charlotte Suppli Ulrik

**Affiliations:** 1 Respiratory Research Unit Hvidovre, Department of Respiratory Medicine, Hvidovre Hospital, 2650 Hvidovre, Denmark; 2 Institute of Clinical Medicine, University of Copenhagen, Copenhagen, Denmark; 3 Værløse Lægehus, Værløse, Denmark; 4 Lægehuset Remisen, Præstø, Denmark

**Keywords:** COPD, ICS, General practice, Exacerbations, Eosinophils

## Abstract

**Background and objective:**

Inhaled corticosteroid (ICS) therapy for COPD should be guided by exacerbations and blood-eosinophils according to the GOLD 2020 strategy document. In the present study, we applied these recent recommendations in a large cohort of COPD patients recruited from general practice.

**Methods:**

The participating general practitioners (n = 144) recruited patients with a diagnosis of COPD currently prescribed ICS and reported data on exacerbation history and blood-eosinophils. Clinical variables were compared using two-sample t-tests.

**Results:**

The study cohort comprised 1,567 COPD patients (44% males and mean age 72 years). In the past 12 months, 849 (54%) of the COPD patients currently prescribed ICS had no exacerbation, whereas 383 (24%) and 328 (21%) patients, respectively, had a history of one exacerbation and two or more exacerbations. Compared to patients with one or no exacerbation, patients with ≥ 2 exacerbations (21%) per year reported more dyspnea (p < 0.001) and had higher degree of airflow obstruction (p < 0.001). Among patients with no and at least one exacerbation within the preceding 12 months, 30% and 26%, respectively, had a blood-eosinophil count ≥ 0.3 × 10^9^/L. In patients with two or more exacerbations within the last 12 months, 77% had a blood-eosinophil count of ≥ 0.1 × 10^9^/L. Furthermore, 166 patients (11%) had at least one hospital admission due to COPD exacerbation, and a blood-eosinophil count of ≥ 0.1 × 10^9^/L.

**Conclusion:**

This study of a large cohort of COPD patients currently prescribed inhaled corticosteroids suggests the need for re-evaluating the management strategy to increase benefit and reduce adverse effects of ICS treatment in COPD patients managed in primary care.

## Introduction

The 2020 strategy document by the Global Initiative for Chronic Obstructive Lung Disease (GOLD) recommends maintenance therapy with inhaled corticosteroids (ICS) together with long-acting bronchodilators for COPD patients with a history of frequent exacerbations despite treatment with long-acting bronchodilators alone [1], as previous studies have shown that the benefit of ICS therapy is greater in patients with high risk of exacerbations [2, 3]. Furthermore, the 2019 report by GOLD states that inhaled maintenance treatment with a combination including ICS improves lung function and health status in COPD patients [4]. However, this combination therapy is often prescribed as an initial treatment regardless of COPD severity [4]. Furthermore, ICS treatment has also been associated with a high risk of pneumonia in COPD patients [5, 6]. It is therefore imperative to limit ICS treatment to COPD patients who are likely to derive benefit therefrom.

Recent studies indicate that patients with elevated blood-eosinophil count have higher risk of COPD exacerbations [7, 8]. Furthermore, a possible association between the number of blood-eosinophils in COPD patients and the effect of ICS on the occurrence of COPD exacerbations has recently been reported [7, 9, 10] with the findings suggesting that COPD patients with the highest blood-eosinophil counts and rate of exacerbation benefit the most from ICS treatment [10].

According to the latest report by GOLD, clinical evaluation of exacerbation risk (≥ 2 exacerbations and/or 1 hospitalization in the previous year) together with blood-eosinophils should be taken into account when prescribing ICS for COPD [1]. According to post hoc analyses of a previous study, treatment regimens containing ICS did not benefit patients with a blood-eosinophil count lower than 100 cells/µL [11]. However, there was a significant favorable effect of ICS treatment in COPD patients with a blood-eosinophil count of more than 300 cells/µL [8]. Other studies support this proposal and conclude that COPD patients with frequent exacerbations and higher blood-eosinophil counts have reduced exacerbations on ICS treatment, though firm conclusions are limited due to arbitrary cut-off of blood-eosinophil count [10, 12].

In this population-based cohort of COPD patients followed in primary care, our aim was to apply recent proposals from the GOLD strategy document, which indicates that ICS therapy should be guided by exacerbations and blood-eosinophils.

## Materials and methods

**Fig. 1:**
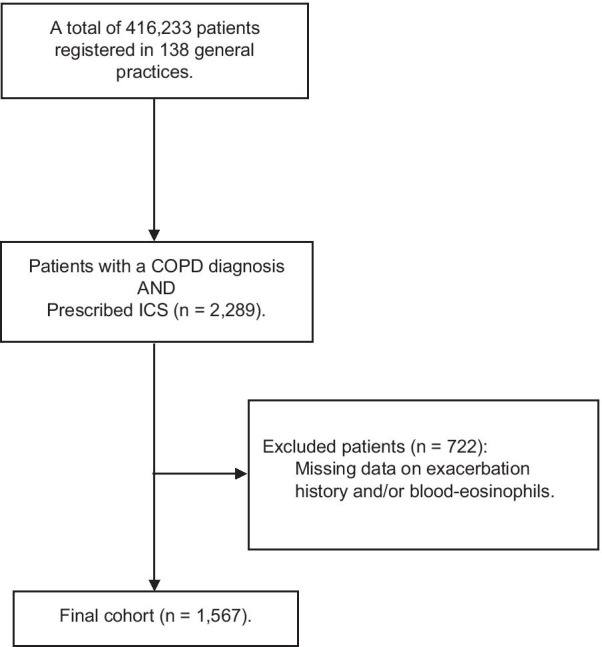
Selection process of patients with chronic obstructive pulmonary disease (COPD) and information on exacerbation rate and/or blood-eosinophils, recruited from primary care and currently prescribed inhaled corticosteroids (ICS)

Participants included in this study had COPD, were prescribed ICS (coded as International Classification of Primary Care, 2nd ed. code R95 in electronic patient journals and with the ACT code R03AK and R03BA, indicating ICS treatment), and managed in primary care. The selection process of patients participating in the study is summarized in Fig. 1, also illustrating that for the present analysis only patients with information on exacerbation rate and/or blood-eosinophils have been included. General practitioner’s (GPs) (n = 144) cross-sectional data were collected in 2017; further details have been published previously [13]. In short, data, if eligible, on demographics, blood-eosinophils, previous COPD exacerbations, characteristics and clinical information were collected from GPs to form a large population-based cohort in Denmark. For inclusion, GPs had to provide a maximum of 20 COPD patients who were currently prescribed ICS. Primary care physicians provided anonymized data with only the specified GP having access to patient identity.

### Definitions

Medical Research Council (MRC) scale was used to assess symptom severity. Blood eosinophil levels were classified as high when ≥ 0.3 × 10^9^/L and low when < 0.1 × 10^9^/L [14]. COPD exacerbations were classified as moderate if the patient had been treated with oral corticosteroid and/or antibiotics out of hospital, with data obtained from the GPs medical records.

### Data analysis

Data were reported as mean values ± one standard deviation (SD). The baseline characteristics of included patients were calculated, and clinical variables were compared between subgroups of patients using independent t-tests for continuous variables (all included variables fulfilled criteria for normal distribution). A p-value < 0.05 was considered statistically significant. Data were analyzed using the statistical program IBM SPSS version 25 (IBM Corporation, Armonk, NY, USA), and data were also entered in Excel (Microsoft, Redmond, WA) spreadsheet for the development of figures.

## Results

### Patient characteristics

Of the COPD patients prescribed ICS recruited from general practice (n = 2,289), 1567 (68%) had complete data on exacerbation history and/or blood-eosinophils from the last 12 months and were, therefore, included in the present analysis; further details are presented in Fig. 1.

In the final cohort of COPD patients, there were more females (56.3%) than males, further baseline characteristics of the included patients are given in Table 1.

**Table 1:** Baseline characteristics of patients with chronic obstructive pulmonary disease (COPD) currently prescribed inhaled corticosteroids (ICS) identified in general practice with complete data on exacerbations and/or blood-eosinophils (*n* = 1567)

	COPD patients (*n* = 1567)	Mean (SD)
Sex		
Females	882 (56.3%)	
Males	685 (43.7%)	
Age (years)	1567 (100%)	71.9 (SD 10.8)
Pack-years^a^	639	33.2 (SD 21.5)
BMI (kg/${\mathrm{m}}^{2}$)	1122	26.9 (SD 6.2)
Symptom score		
MRC-score		
≤ 2	368 (23.5%)	
≥ 3	434 (27.7%)	
Spirometry		
FEV_1_ %pred (%)	1249 (79.7%)	60.0 (SD 23.8)
FEV_1_ (L)	1264 (80.7%)	1.51 (SD 0.66)
FEV_1_/FVC	1329 (84.8%)	0.58 (SD 0.15)

*COPD* chronic obstructive pulmonary disease, *BMI* body mass index, *MRC* Medical Research Council, *FEV*_*1*_ forced expiratory volume in 1. second, *FVC* forced vital capacity, *SD* standard deviation

^a^Pack-years include current smokers and ex-smokers

### Exacerbation history

**Fig. 2:**
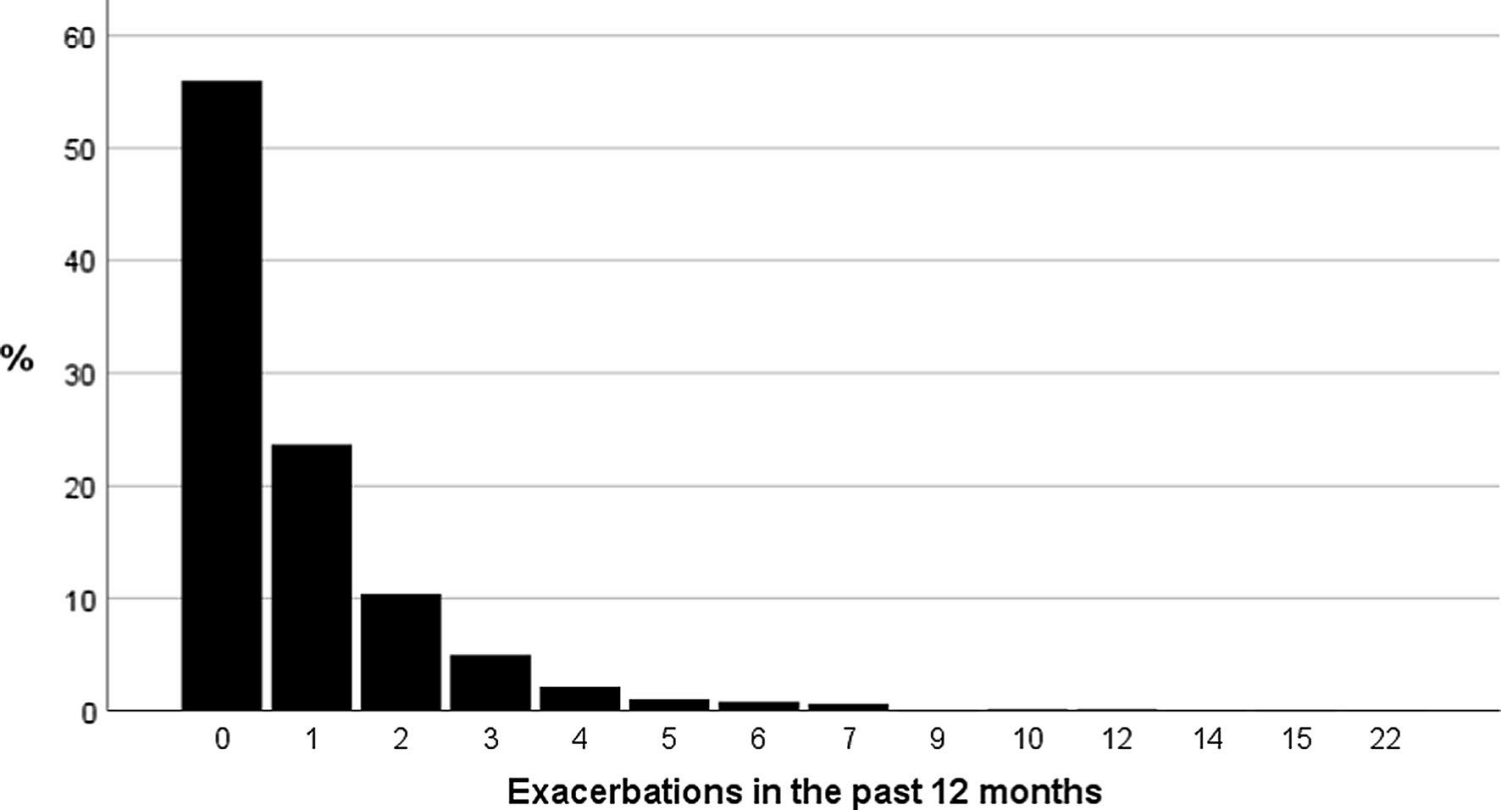
Patients with chronic obstructive pulmonary disease (COPD) recruited from primary care and currently prescribed inhaled corticosteroids (ICS) (n = 1567) stratified according to frequency of exacerbations (0, 1 or ≥ 2 annual exacerbations)

Of the enrolled patients, 20.9% (n = 328) had a history of two or more exacerbations in the preceding year with 14.9% (n = 234) of all patients having had at least one hospital admission for COPD within the last 12 months. As shown in Fig. 2, 54% of patients had not had any exacerbations in the last 12 months. Further details on exacerbation rate is given in Fig. 2.

Compared to patients with < 2 exacerbations patients with ≥2 exacerbations (21%) per year had higher MRC-score (p<0.001) and lower FEV_1_/FVC (p < 0.001). Further details are presented in Tables 2 and 3.

**Table 2:** Characteristics associated with chronic obstructive pulmonary disease (COPD) patients (*n* = 1567) identified in general practice and currently in inhaled corticosteroid (ICS) treatment stratified by frequency of exacerbations (0, 1 or ≥ 2 annual exacerbations)

	0 exacerbations per year (*n* = 849)	1 exacerbation per year (*n* = 383)	≥ 2 exacerbations per year (*n* = 328)
BMI (kg/${\mathrm{m}}^{2}$), mean	27.15 (SD 5.99)	27.17 (SD 6.12)	26.28 (SD 6.76)
Pack-years, mean	30.49 (SD 20.89)	36.04 (SD 22.97)	36.90 (SD 20.45)
${\mathrm{FEV}}_{1}/\mathrm{FVC}$, mean	0.60 (SD 0.14)	0.57 (SD 0.15)	0.53 (SD 0.15)
${\mathrm{FEV}}_{1}$%predicted (%), mean	64.71 (SD 23.52)	57.10 (SD 22.58)	51.43 (SD 22.99)
${\mathrm{FEV}}_{1}$(L), mean	1.64 (SD 0.66)	1.43 (SD 0.62)	1.27 (SD 0.64)
B-eosinophils (10^9^/L), mean	0.25 (SD 0.21)	0.25 (SD 0.22)	0.23 (SD 0.19)
MRC-score, mean	2.38 (SD 1.02)	2.82 (SD 1.11)	3.20 (SD 1.04)

*BMI* body mass index, *FEV*_*1*_ forced expiratory volume in 1. second, *FVC* forced vital capacity, *B-eosinophils* blood-eosinophils, *MRC* medical research council, *SD* standard deviation

**Table 3:** Prevalence of variables in included cohort

	< 2 exacerbation per year (*n* = 1230)	≥ 2 exacerbations per year (*n* = 327)	P- value
BMI (kg/m^2^), *n*	890 (27.16)	229 (26.28)	0.55
Pack-years, *n*	501 (32.24)	134 (36.90)	0.26
FEV_1_/FVC, *n*	1007 (0.59)	253 (0.53)	*< 0.001*
FEV_1_ %predicted (%), *n*	980 (62.37)	265 (51.43)	*< 0.001*
FEV_1_ (L), *n*	1052 (1.57)	271 (1.27)	*< 0.001*
B-eosinophils (10^9^/L), *n*	1232 (0.25)	328 (0.23)	0.17
MRC-score, *n*	604 (2.53)	196 (3.20)	*< 0.001*

*BMI* body mass index, *FEV*_*1*_ forced expiratory volume in 1. second, *FVC* forced vital capacity, *B-eosinophils* blood-eosinophils, *MRC* medical research council

P-values less than 0.05 are considered statistically significant (displayed in italic text) and calculated for the difference between the subgroup < 2 and ≥ 2 exacerbations per year

### Exacerbations in COPD patients in relation to blood eosinophils

**Fig. 3:**
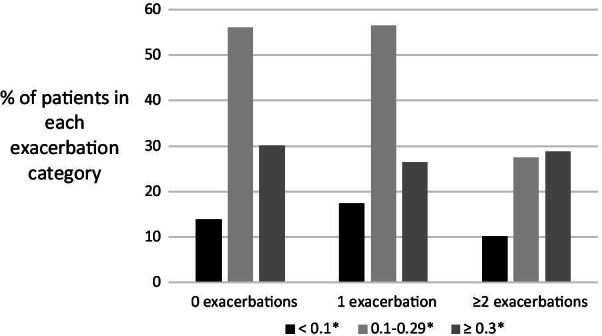
Patients with chronic obstructive pulmonary disease (COPD) recruited from primary care and currently prescribed inhaled corticosteroids (ICS) (n = 1567) stratified according to annual number of exacerbations (0, 1 or ≥ 2) and blood-eosinophil count

Among patients with no exacerbations within the preceding 12 months (n = 849), 30% had a blood-eosinophil count ≥ 0.3 × 10^9^/L, compared to 26% (n = 101) in patients with a history of at least one exacerbation. In patients with two or more exacerbations within the last 12 months, 77% had a blood-eosinophil count of ≥ 0.1 × 10^9^/L, and, furthermore, 166 patients (10.6%) had at least one exacerbation, at least one hospital admission due to COPD exacerbation, and a blood-eosinophil count of ≥ 0.1 × 10^9^/L. Further details on frequency of exacerbations in relation to blood-eosinophils are given in Fig. 3.

### Treatment for COPD exacerbations according to blood eosinophils

In those with a blood-eosinophil count ≤ 0.1 × 10^9^/L and a minimum of two moderate exacerbations within the last year (n = 91), 17 patients were more likely to be treated with systemic corticosteroids than antibiotics, while 33 patients were prescribed more antibiotics than corticosteroids the preceding 12 months. In this group of patients, 49 patients (54%) were treated with long-acting muscarinic antagonists (LAMA).

In those with a blood-eosinophil count ≥ 0.3 × 10^9^/L and a minimum of two moderate exacerbations within the last year (n = 88), 13 patients had more treatments with corticosteroids than antibiotics, while 44 (50%) patients were prescribed more antibiotics than corticosteroids within the last year. In this group of patients, 42 patients were treated with LAMA.

## Discussion

The present study of a large cohort of COPD patients managed in primary care and currently prescribed ICS showed that 54% of the patients had no exacerbation within the last 12 months, whereas 21% had a history of two or more exacerbations. Among patients with no history of exacerbations, 30% had a blood-eosinophil count ≥ 0.3 × 10^9^/L.

Contrary to the strategy document by GOLD, which provides guidelines for physicians on when to consider ICS treatment, the Danish Society of Respiratory Medicine provides recommendations on de-escalation of ICS maintenance therapy in COPD patients without exacerbations or hospitalizations for at least one year due to COPD [15]. In short, a physician may consider halve the dose of ICS and await results for 3 months. If FEV_1_ is more than fifty percent complete withdrawal of ICS may happen followed by a follow-up 3 months later for lung function measurement and assessment of clinical condition. These recommendations may also apply to COPD patients, who have been in ICS and LABA treatment for a long period of time without clear indication.

Consideration of ICS add-on maintenance treatment can be made, according to GOLD, based on exacerbations and symptoms. More specifically in those with a blood-eosinophil count of ≥ 0.1 × 10^9^/L and a history of two or more moderate exacerbations or a blood-eosinophil count of ≥ 0.3 × 10^9^/L [1]. These recommendations facilitate the use of ICS treatment for the prevention of exacerbations in accordance with recent clinical trials presented by a recent post-hoc analysis, which regards blood-eosinophils as a determinant of the benefit of ICS in preventing future COPD exacerbations and presents results indicating a greater benefit of ICS in patients with higher eosinophil count [11]. Almost no effect was reported in those with a blood-eosinophil count less than 0.1 × 10^9^/L, which naturally has been the threshold where patients are most unlikely to benefit from ICS maintenance treatment [11]. Conversely, patients with a blood-eosinophil count of ≥ 0.3 × 10^9^/L have the most benefit from ICS treatment [9]. However, the idea of blood-eosinophils being a biomarker for exacerbation risk is insufficient. Studies have found that blood-eosinophils have less likelihood in determining the future exacerbation risk [16]. Our findings indicate that a substantial proportion of COPD patients prescribed ICS are likely not to benefit from this treatment, as almost one-fifth of the included COPD patients had no exacerbations and blood-eosinophils of less than 0.1 × 10^9^/L and were hence not candidates for ICS maintenance therapy according to guidelines.

This study also assessed whether exacerbation treatment had a correlation to blood-eosinophil count in COPD patients in primary care. Our results indicated that prescription of ICS in patients with two or more moderate exacerbations was made despite a blood-eosinophil count < 0.1 × 10^9^/L. Our study found no correlation between blood-eosinophil count and differences in treatment with corticosteroids and/or antibiotics in COPD exacerbation. According to GOLD, ICS treatment can be considered in patients with two or more moderate exacerbations of COPD per year, while blood-eosinophils < 0.1 × 10^9^/L is an argument against ICS treatment [14]. Moreover, a treatment strategy including LAMA/LABA is preferred in patients with a blood-eosinophil count ≤ 0.1 × 10^9^/L, while LABA/ICS has proven more effective in patients with high blood-eosinophil counts (> 0.3 × 10^9^/L) [17]. However, we found that the proportion of patients prescribed LAMA was only slightly different between patients with a blood-eosinophil count ≤ 0.1 × 10^9^/L and > 0.3 × 10^9^/L, respectively (n = 49 vs 42). On the other hand, if patients experience repeated exacerbations despite appropriate long-acting bronchodilator treatment, add-on treatment with ICS may be considered [14].

**Fig. 4:**
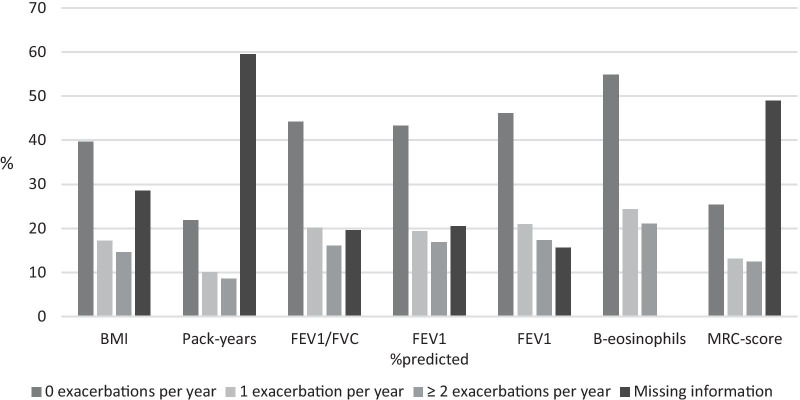
Percentage of included COPD patients (n = 1567) distributed according to variables and stratified by exacerbations per year. *BMI* body mass index, *FEV*_*1*_ forced expiratory volume in 1. second, *FVC* forced vital capacity, *B-eosinophils* blood-eosinophils, *MRC* medical research council

**Fig. 5:**
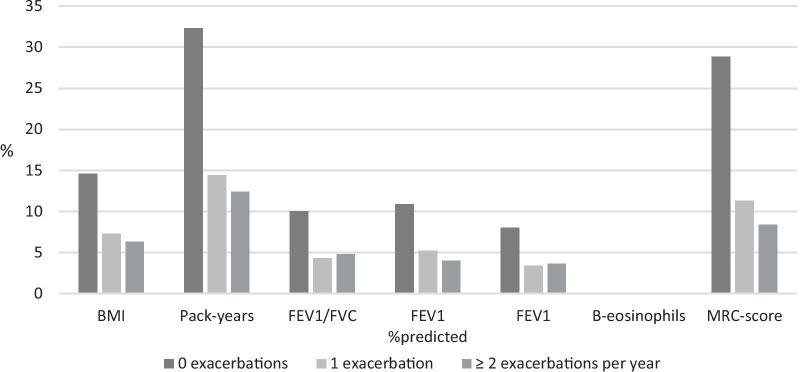
Distribution of missing information in included COPD patients (n = 1567) across variables stratified by exacerbations per year. *BMI* body mass index, *FEV*_*1*_ forced expiratory volume in 1. second, *FVC* forced vital capacity, *B-eosinophils* blood-eosinophils, *MRC* medical research council.

Some limitations are worth mentioning in this study. First, this analysis did not include information on exacerbations leading to hospital admittance defined as severe exacerbations. Second, among all patient data provided by GPs only 1567 COPD patients were included due to missing data on blood-eosinophils and/or exacerbation history. This might have over- or underestimated the prevalence of exacerbations and altered the results. In addition, a few variables in this study had rather considerable missing information (Fig. 4). One could argue whether variables with much missing information are comparable to each other. In this study missing information in most variables is almost equally distributed across the groups compared, which makes comparison of groups feasible. This is, however, not the case for the variables MRC-score and pack-years, which may have distorted results. Further details are given in Fig. 5.

A significant correlation between patients with higher blood-eosinophil count and increased risk of exacerbations has previously been proposed indicating that patients might have higher blood-eosinophil count with increasing exacerbation frequency. However, this was not the case in our study [8]. Furthermore, COPD patients in ICS treatment followed in primary care were enrolled in 2017 prior to the release of the newest strategy document by GOLD. This analysis might be considered incomprehensive in the investigation of an endpoint such as general practitioner’s use of the GOLD report on exacerbations and blood-eosinophils to guide the prescription of ICS. One could argue on the validity of the application of recent recommendations by GOLD on this population-based large cohort of COPD patients currently prescribed ICS. Nevertheless, this analysis gives an initial retrospective assessment on the ICS prescription pattern in general practice.

There is a need for future studies to evaluate whether primary care physicians have changed ICS prescription behavior due to recent GOLD strategy document. The recent studies pointing to a more beneficial treatment regimen guided by exacerbations and blood-eosinophils could alter the risk-benefit ratio by reducing future incidence of ICS adverse effects and potentially promote the reduction of mortality and COPD-related morbidity in ICS prescribed COPD patients in general practice. Further research is needed to determine to what extent ICS maintenance therapy, in accordance with the recent strategy report by GOLD, benefit COPD patients managed in general practice, and by that, presumably, have less severe disease, as this may pave the way for a more personalized approach to the management of COPD, also in general practice.

## Data Availability

The database will be, upon request, be available from the corresponding author according to current legislation.

## Abbreviations

COPD: Chronic obstructive pulmonary disease
FEV1: Forced expiratory volume in 1. second
FVC: Forced vital capacity
GOLD: Global initiative for chronic obstructive lung disease
GP: General practitioner
ICS: Inhaled corticosteroids
MRC: Medical research council
